# Camrelizumab-based therapies for the treatment of advanced lung cancer: a prospective, open-label, multicenter, observational, real-world study

**DOI:** 10.3389/fimmu.2025.1494708

**Published:** 2025-03-07

**Authors:** Dong Zhao, Minghong Bi, Xiaofei Cheng, Shuhong Wang, Huaidong Cheng, Xiaoyang Xia, Huan Chen, Yanbei Zhang, Zhiqiang Hu, Qisheng Cao, Hui Liang, Fan Wang, Xuhong Min, Ling Xu, Kehai Feng, Jinhua Zhou, Xinzhong Li, Rui Wang, Hua Xie, Xiaosi Chen, Kangsheng Gu

**Affiliations:** ^1^ Department of Oncology, Lixin County People’s Hospital, Bozhou, China; ^2^ Department of Oncology, The First Affiliated Hospital of Bengbu Medical College, Bengbu, China; ^3^ Department of Oncology, Anqing Hospital of Navy, Anqing, China; ^4^ Department of Oncology, Huangshan City People’s Hospital, Huangshan, China; ^5^ Department of Oncology, The Second Affiliated Hospital of Anhui Medical University, Hefei, China; ^6^ Department of Oncology, Chuzhou City First People’s Hospital, Chuzhou, China; ^7^ Department of Pulmonary and Critical Care Medicine, Anqing Hospital of Navy, Anqing, China; ^8^ Department of Pulmonary and Critical Care Medicine, The First Affiliated Hospital of Anhui Medical University, Hefei, China; ^9^ Department of Oncology, Huaibei Miner General Hospital, Huaibei, China; ^10^ Interventional Oncology, Maanshan City People’s Hospital, Maanshan, China; ^11^ Department of Radiology, Traditional Chinese Hospital of LuAn, Luan, China; ^12^ Department of Radiology, The First Affiliated Hospital of Anhui Medical University, Hefei, China; ^13^ Department of Radiology, Anhui Chest Hospital, Hefei, China; ^14^ Department of Pulmonary and Critical Care Medicine, Anhui Chest Hospital, Hefei, China; ^15^ Department of Oncology, The First Affiliated Hospital of USTC West District, Hefei, China; ^16^ Department of Oncology, The First Affiliated Hospital of Anhui University of Traditional Chinese Medicine, Hefei, China; ^17^ Department of Oncology, Huaibei City People’s Hospital, Huaibei, China; ^18^ Department of Oncology, Anhui Chest Hospital, Hefei, China; ^19^ Department of Oncology, Xuancheng City People’s Hospital, Xuancheng, China; ^20^ Department of Oncology, Dangtu County People’s Hospital, Maanshan, China; ^21^ Department of Oncology, The First Affiliated Hospital of Anhui Medical University, Hefei, China

**Keywords:** advanced lung cancer, camrelizumab-based therapies, treatment response, survival, adverse events

## Abstract

**Objective:**

Camrelizumab, a programmed death-1 inhibitor, is effective and safe for treating patients with advanced lung cancer according to previous phase 3 trials. However, relevant real-world clinical evidence is required. This study intended to explore the efficacy and safety of camrelizumab-based therapies in patients with advanced lung cancer.

**Methods:**

Patients with advanced lung cancer who received camrelizumab-based therapies as first-line or above treatment were consecutively enrolled in this study. The median follow-up duration was 5 months.

**Results:**

A total of 298 subjects were enrolled. Objective response rate (ORR) and disease control rate (DCR) were 27.2% and 82.2%. Multivariable logistic regression analysis showed that previous pulmonary surgery [odds ratio (OR)=0.440, *P*=0.024], previous radiotherapy (OR=0.410, *P*=0.010), and Eastern Cooperative Oncology Group Performance Status (ECOG PS) score (>1 vs. 0~1) (OR=0.414, *P*=0.046) were independently and negatively associated with ORR. The median progression-free survival (PFS) [95% confidence interval] was 10.0 (7.8-12.2) months. Median overall survival (OS) was not reached. Multivariable Cox regression analysis suggested that brain metastasis [hazard ratio (HR)=1.548, *P*=0.036] and liver metastasis (HR=1.733, *P*=0.035) were independently associated with shorter PFS. Previous chemotherapy (HR=2.376, *P*=0.022), brain metastasis (HR=2.688, *P*=0.006), and liver metastasis (HR=2.583, *P*=0.039) were independently associated with shorter OS. Most adverse events were grade I or II. Grade III and IV adverse events rarely occurred. The occurrence of adverse events was associated with a higher DCR (*P*=0.003).

**Conclusions:**

Camrelizumab-based therapies may serve as potential treatments for patients with advanced lung cancer. However, further studies with an extended follow-up duration are warranted.

## 1 Introduction

Lung cancer, the most frequently diagnosed cancer, contributes to 2.5 million new cases and 1.8 million cancer-related deaths worldwide in 2022 (1). Lung cancer can be classified into two categories, including non-small cell lung cancer (NSCLC) and small cell lung cancer (SCLC), comprising nearly 85% and 15% of all lung cancer cases, respectively (2, 3). Surgery remains the cornerstone of early-stage lung cancer, but most patients are diagnosed in the advanced stage (3, 4). Despite the advancements in the treatments for advanced lung cancer, the prognosis of these patients is unsatisfactory, with a 5-year relative survival rate of 6% to 33% (5). Therefore, exploring potential treatments is meaningful to improve the prognosis of patients with advanced lung cancer.

The emergence of immune checkpoint inhibitors (ICIs), such as programmed death-1 (PD-1) inhibitors, has revolutionized the therapeutic landscape of advanced lung cancer (6, 7). PD-1 inhibitors exert their function by blocking the interaction between PD-1 and its primary ligand, programmed death-ligand 1 (PD-L1), which enables T cells to recognize and attack cancer cells (7, 8). Recently, it has been found that several PD-1 inhibitors, such as nivolumab (9–12), cemiplimab (13, 14), and pembrolizumab (15–18), used alone or combined with chemotherapy, targeted therapy, or radiotherapy, achieve good efficacy with tolerable safety in patients with advanced lung cancer.

Camrelizumab, a humanized monoclonal antibody against PD-1, possesses antitumor activity with an acceptable safety profile in multiple advanced cancers (19–23). Regarding advanced lung cancer, previous studies suggested that camrelizumab-based therapies could serve as the first-line treatment for this disease (21, 23). The CameL study reported that first-line camrelizumab plus carboplatin and pemetrexed achieved a median progression-free survival (PFS) of 11.3 months in patients with advanced non-squamous NSCLC (23). The CameL-Sq study observed that the objective response rate (ORR) and disease control rate (DCR) were 64.8% and 88.1% in patients with advanced squamous NSCLC who received first-line camrelizumab plus carboplatin and paclitaxel (21). Nevertheless, most of the existing studies focus on the potential of first-line camrelizumab-based therapies, but their role as a second or subsequent-line treatment for advanced lung cancer deserves to be explored. Also, relevant real-world evidence is required.

Accordingly, this real-world study enrolled patients with advanced lung cancer who received camrelizumab-based therapies as first-line or above treatment, aiming to investigate the efficacy and safety of camrelizumab-based therapies in these patients.

## 2 Methods

### 2.1 Patients

In this prospective, open-label, multicenter, observational study, 298 patients with advanced lung cancer were consecutively enrolled from August 2019 to February 2021. The inclusion criteria were: 1) diagnosed with lung cancer by histological or cytological method; 2) with the IIIB to IV stage of disease; 3) aged more than 18 years old; 4) received camrelizumab as first-line or above treatment; 5) joined this study voluntarily and signed an informed consent form; 6) could benefit from treatment. The exclusion criteria were: 1) with other primary solid cancers or hematological malignancies; 2) with a proven allergy to the experimental drug and/or its excipients; 3) with immunodeficiency diseases; 4) with a history of organ transplantation; 5) pregnant or lactating women. This study received approval from the Ethics Committee. The signed informed consent was collected from each patient. The Clinical Trial Registration number was ChiCTR2000034595.

### 2.2 Camrelizumab-based therapy

This study did not interfere with the medication of patients. The regimens were determined according to patients’ own conditions, patients’ own willingness, and doctors’ advice. The types of camrelizumab-based therapies were briefly described as follows: 1) camrelizumab monotherapy, 2) camrelizumab plus chemotherapy, 3) camrelizumab plus targeted therapy, 4) camrelizumab plus chemotherapy and targeted therapy, 5) camrelizumab plus chemotherapy and radiotherapy, 6) camrelizumab plus radiotherapy. The dosage of camrelizumab was 200 mg, which was administered once every 3 weeks via intravenous infusion on the 1^st^ day. During the study period, the administration of medication could be paused, reduced, or discontinued due to the side effects. Treatment was continued until the disease progressed. The targeted therapy, chemotherapy, or radiotherapy administration was based on the actual condition of the patients and the clinical experience of the investigators.

### 2.3 Data collection and evaluation

Demographics and disease-related information were collected. The image examination data were also collected, and based on them, the efficacy was evaluated every 2 cycles by Response Evaluation Criteria in Solid Tumors (v.1.1) (RECIST 1.1) (24). The best clinical response was evaluated and documented. Using these responses, the ORR and DCR were computed.

### 2.4 Follow-up

Patients underwent routine follow-ups with a median value of 5 months. PFS was defined as the duration between the start of treatment and disease progression or any-cause mortality. Overall survival (OS) was defined as the duration between the start of treatment and any-cause mortality. Adverse events were also captured, which were evaluated via the Common Terminology Criteria for Adverse Events (v.5.0). The primary endpoint was PFS. The secondary endpoints were OS, ORR, DCR, and adverse events.

### 2.5 Statistics

The data analysis was conducted using SPSS v.26.0 software from IBM, USA. Univariable and forward-stepwise method multivariable logistic regression models were conducted to explore factors associated with ORR. Kaplan-Meier curves were used for survival analyses, and the Log-rank test was used to compare survival between different subgroups. Univariable and forward-stepwise method multivariable Cox regression models were built to explore factors that influenced PFS and OS independently. The forward-stepwise logistic or Cox regression method was used to incorporate all the variables in the univariate analysis into the multivariate model to find the independent influencing factors. Sites of metastases had a very strong association with lymph node metastasis, bone metastasis, brain metastasis, liver metastasis, and pleura metastasis; besides, treatment lines had a very strong association with previous chemotherapy. Therefore, to reduce multicollinearity, sites of metastases and treatment lines are not included in the multivariable analyses. The *χ* ^2^ test or Fisher’s exact test was applied to compare ORR and DCR between groups. A *P* value <0.05 (two-sided) indicated significance.

## 3 Results

### 3.1 Clinical features and therapy information

The mean age of patients was 63.7 ± 10.9 years. Sixty-three (21.1%) patients were female and 235 (78.9%) patients were male. There were 248 (83.2%) patients with an Eastern Cooperative Oncology Group Performance Status (ECOG PS) score of 0~1 and 50 (16.8%) patients with an ECOG PS score of >1. Forty-eight (16.1%), 6 (2.0%), and 244 (81.9%) patients had a tumor-node-metastasis (TNM) stage of IIIB, IIIC, and IV, respectively. Ninety-three (31.2%) patients received camrelizumab-based therapies as first-line treatment, and the remaining 205 (68.8%) patients received camrelizumab-based therapies as second or subsequent-line treatment. Detailed information of patients is shown in **Table 1**.

**Table 1:** Clinical characteristics of patients with advanced lung cancer.

Characteristics	Patients (N = 298)
Age (years), mean ± SD	63.7 ± 10.9
Gender, n (%)	
Female	63 (21.1)
Male	235 (78.9)
Smoking history, n (%)	73 (24.5)
Drinking history, n (%)	35 (11.7)
Previous lung diseases, n (%)	14 (4.7)
Previous pulmonary surgery, n (%)	65 (21.8)
Previous chemotherapy, n (%)	205 (68.8)
Previous radiotherapy, n (%)	85 (28.5)
Previous ICI therapy, n (%)	15 (5.0)
ECOG PS score, n (%)	
0~1	248 (83.2)
>1	50 (16.8)
Histological subtype, n (%)	
Adenocarcinoma	127 (42.6)
Squamous cell carcinoma	108 (36.2)
Small-cell carcinoma	39 (13.1)
Others or UK	24 (8.1)
Sites of metastases, n (%)	
None	66 (22.1)
1~2	167 (56.0)
>2	62 (20.8)
UK	3 (1.0)
Location of metastasis, n (%)	
Lymph node metastasis	135 (45.3)
Bone metastasis	59 (19.8)
Brain metastasis	45 (15.1)
Liver metastasis	28 (9.4)
Pleura metastasis	19 (6.4)
TNM stage, n (%)	
IIIB stage	48 (16.1)
IIIC stage	6 (2.0)
IV stage	244 (81.9)
*EGFR* mutation-positive, n (%)	31 (10.4)
*ALK-*positive, n (%)	4 (1.3)
*ROS-1-*positive, n (%)	4 (1.3)
Treatment lines, n (%)	
First	93 (31.2)
Second or above	205 (68.8)

SD, standard deviation; ICI, immune checkpoint inhibitors; ECOG PS, Eastern Cooperative Oncology Group Performance Status; UK, unknown; TNM, tumor-node-metastasis; EGFR, epidermal growth factor receptor; ALK, anaplastic lymphoma kinase; ROS-1, Ros proto-oncogene 1.

Regarding therapy information, 186 (62.4%) patients received camrelizumab combined with chemotherapy and 71 (23.8%) patients received camrelizumab combined with targeted therapy. The detailed therapy information is exhibited in **Table 2**.

**Table 2:** Information on camrelizumab-based therapy.

Items	Patients (N = 298)
Details, n (%)	
Camrelizumab monotherapy	53 (17.8)
Camrelizumab plus chemotherapy	171 (57.4)
Camrelizumab plus targeted therapy	58 (19.5)
Camrelizumab plus chemotherapy and targeted therapy	13 (4.4)
Camrelizumab plus chemotherapy and radiotherapy	2 (0.7)
Camrelizumab plus radiotherapy	1 (0.3)
Summary, n (%) ^‡^	
Camrelizumab combined with chemotherapy	186 (62.4)
Camrelizumab combined with targeted therapy	71 (23.8)

‡ Special illustration:

‘Camrelizumab combined with chemotherapy’ contained ‘camrelizumab plus chemotherapy’, ‘camrelizumab plus chemotherapy and targeted therapy’, and ‘camrelizumab plus chemotherapy and radiotherapy’;

‘Camrelizumab combined with targeted therapy’ contained ‘camrelizumab plus targeted therapy’ and ‘camrelizumab plus chemotherapy and targeted therapy’.

### 3.2 Clinical response

**Figure 1:**
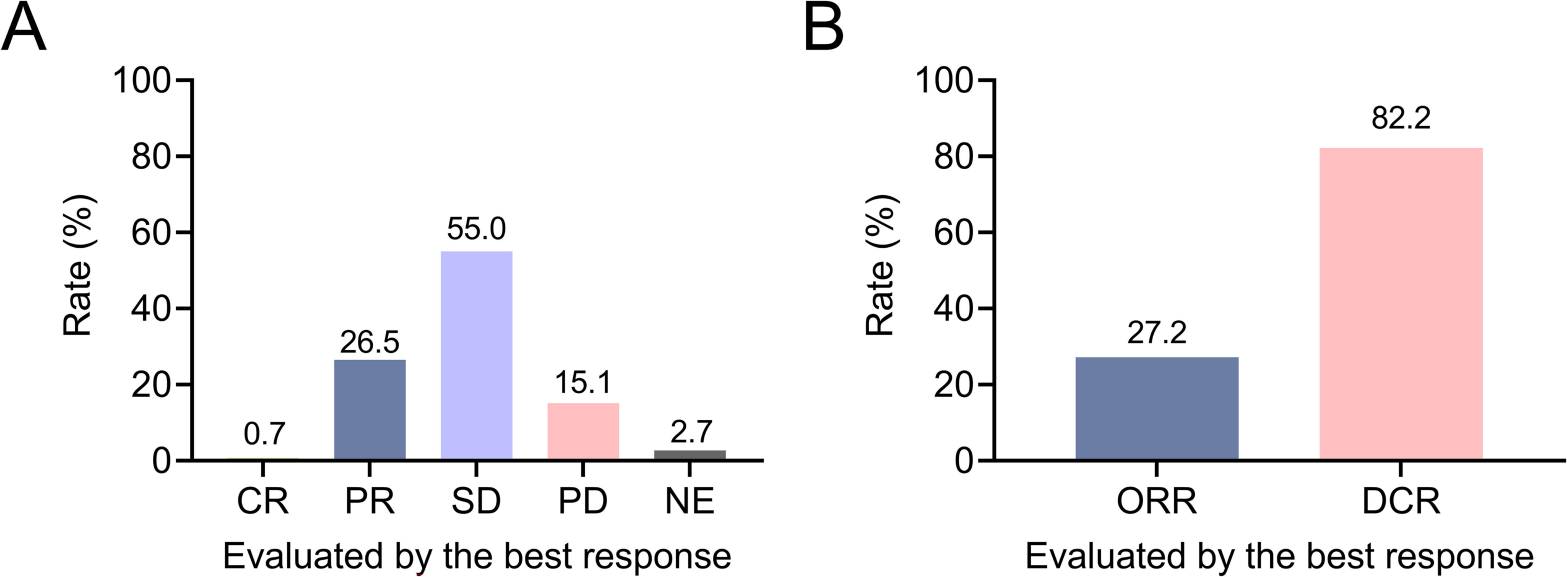
Exhibition of clinical response in patients with advanced lung cancer. Rates of CR, PR, SD, PD, and NE **(A)**; ORR and DCR **(B)** in patients with advanced lung cancer.

The rates of complete response (CR), partial response (PR), stable disease (SD), and progressive disease (PD) were 0.7%, 26.5%, 55.0%, and 15.1%, respectively; the clinical response was not evaluated (NE) in 2.7% of patients (**Figure 1A**). ORR was 27.2% and DCR was 82.2% in patients with advanced lung cancer (**Figure 1B**). In patients who received first-line camrelizumab-based therapies, ORR and DCR were 36.6% and 86.0%. In patients who received second or subsequent-line camrelizumab-based therapies, ORR and DCR were 22.9% and 80.5%.

### 3.3 Association of driver genes with clinical response

*Epidermal growth factor receptor (EGFR)* mutation-positive, *anaplastic lymphoma kinase (ALK)*-positive, and *Ros proto-oncogene 1 (ROS-1)*-positive were not related to ORR or DCR (all *P*>0.05) (**Supplementary Table 1**).

### 3.4 Logistic regression model for ORR

Univariable logistic regression analysis showed that age (≥65 years vs. <65 years) was positively associated with ORR [odds ratio (OR)=1.882, *P*=0.018]. Previous chemotherapy (OR=0.516, *P*=0.015), previous radiotherapy (OR=0.385, *P*=0.004), ECOG PS score (>1 vs. 0~1) (OR=0.383, *P*=0.026), and bone metastasis (OR=0.418, *P*=0.025) were negatively associated with ORR. According to multivariable logistic regression analysis, previous pulmonary surgery (OR=0.440, *P*=0.024), previous radiotherapy (OR=0.410, *P*=0.010), and ECOG PS score (>1 vs. 0~1) (OR=0.414, *P*=0.046) were independently and negatively associated with ORR (**Table 3**).

**Table 3:** Univariable and multivariable analyses through Logistic regression model for ORR.

Characteristics	*P* value	OR	Lower limit of 95%CI	Upper limit of 95%CI
Univariable analysis				
Age (≥65 years vs. <65 years)	0.018	1.882	1.114	3.177
Gender (male vs. female)	0.218	0.686	0.376	1.250
Smoking history (yes vs. no)	0.962	1.015	0.561	1.835
Drinking history (yes vs. no)	0.836	0.918	0.411	2.054
Previous lung diseases (yes vs. no)	0.622	0.720	0.196	2.651
Previous pulmonary surgery (yes vs. no)	0.077	0.538	0.271	1.069
Previous chemotherapy (yes vs. no)	0.015	0.516	0.303	0.880
Previous radiotherapy (yes vs. no)	0.004	0.385	0.200	0.743
Previous ICI therapy (yes vs. no)	0.584	1.362	0.451	4.113
ECOG PS score (>1 vs. 0~1)	0.026	0.383	0.165	0.890
Histological subtype				
Adenocarcinoma (reference)	Reference	(-)	(-)	(-)
Squamous cell carcinoma vs. reference	0.100	1.617	0.912	2.866
Small-cell carcinoma vs. reference	0.944	0.970	0.415	2.269
Others or UK vs. reference	0.885	1.078	0.392	2.961
Lymph node metastasis (yes vs. no)	0.261	1.341	0.804	2.237
Bone metastasis (yes vs. no)	0.025	0.418	0.195	0.894
Brain metastasis (yes vs. no)	0.655	0.846	0.406	1.761
Liver metastasis (yes vs. no)	0.051	0.295	0.087	1.007
Pleura metastasis (yes vs. no)	0.332	1.616	0.613	4.260
TNM stage				
IIIB (reference)	Reference	(-)	(-)	(-)
IIIC vs. reference	0.375	0.365	0.039	3.382
IV vs. reference	0.175	0.635	0.329	1.225
*EGFR* mutation-positive (yes vs. no)	0.503	1.315	0.590	2.927
*ALK-*positive (yes vs. no)	0.069	8.308	0.852	81.054
*ROS-1-*positive (yes vs. no)	0.069	8.308	0.852	81.054
Camrelizumab combined with chemotherapy (yes vs. no)	0.233	1.389	0.809	2.383
Camrelizumab combined with targeted therapy (yes vs. no)	0.315	0.725	0.387	1.357
Multivariable analysis				
Previous pulmonary surgery (yes vs. no)	0.024	0.440	0.216	0.900
Previous radiotherapy (yes vs. no)	0.010	0.410	0.208	0.809
ECOG PS score (>1 vs. 0~1)	0.046	0.414	0.174	0.984
Bone metastasis (yes vs. no)	0.051	0.458	0.209	1.005
*ALK-*positive (yes vs. no)	0.097	7.164	0.701	73.199

ORR, objective response rate; OR, odds ratio; CI, confidence interval; ICI, immune checkpoint inhibitors; ECOG PS, Eastern Cooperative Oncology Group Performance Status; UK, unknown; TNM, tumor-node-metastasis; EGFR, epidermal growth factor receptor; ALK, anaplastic lymphoma kinase; ROS-1, Ros proto-oncogene 1.

‘Sites of metastases’ and ‘Treatment lines’ were not included in the model: ‘Sites of metastases’ had a very strong association with ‘Lymph node metastasis,’ ‘Bone metastasis,’ ‘Brain metastasis,’’ Liver metastasis,’ ‘Pleura metastasis’; and ‘Treatment lines’ had a very strong association with ‘Previous chemotherapy’.

### 3.5 Survival profiles

**Figure 2:**
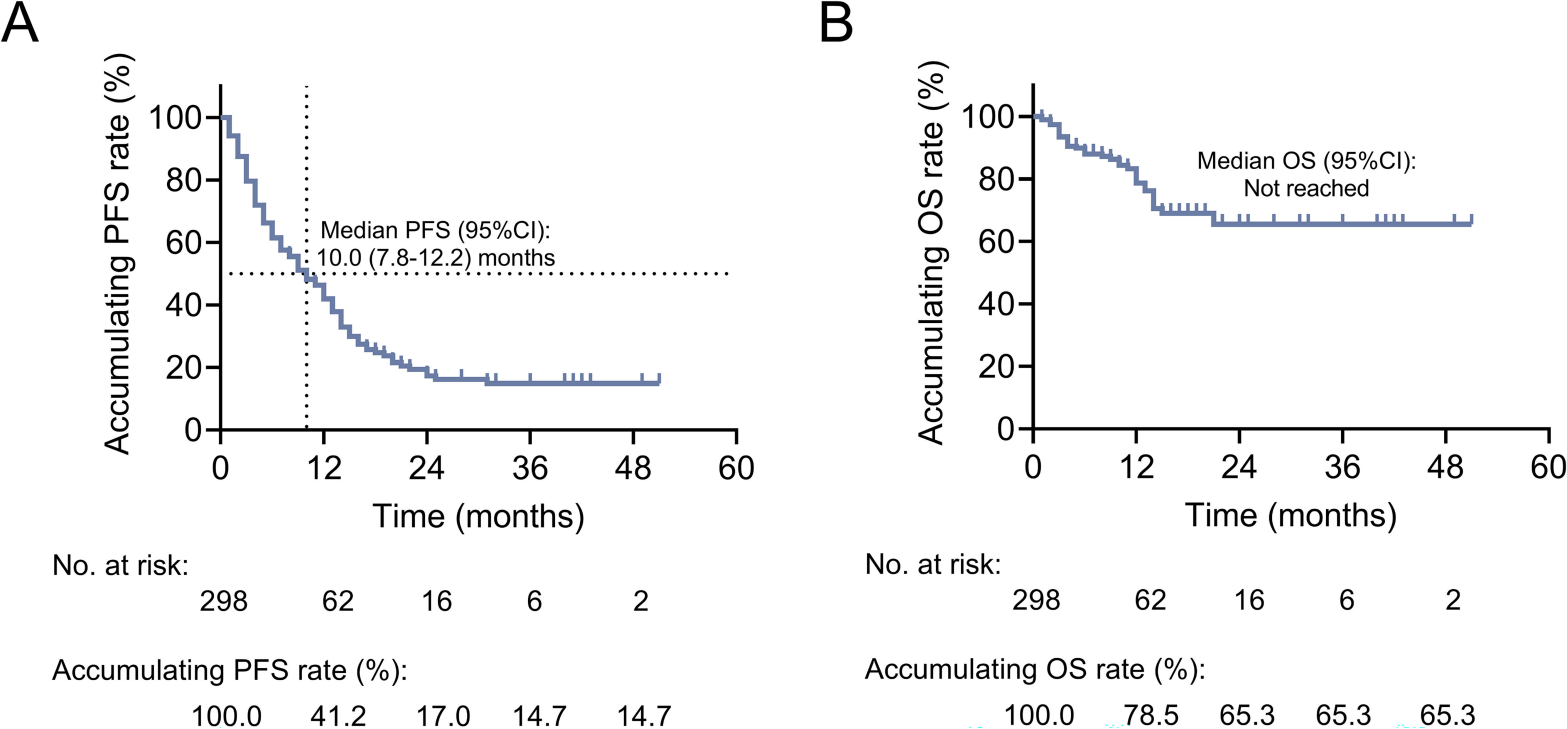
Exhibition of survival profiles in patients with advanced lung cancer. Kaplan-Meier curves of PFS **(A)** and OS **(B)** in patients with advanced lung cancer.

The median PFS [95% confidence interval (CI)] was 10.0 (7.8-12.2) months. The 12-, 24-, 36-, and 48-month accumulating PFS rates were 41.2%, 17.0%, 14.7%, and 14.7%, respectively (**Figure 2A**). The median OS was not reached. The 12-, 24-, 36-, and 48-month accumulating OS rates were 78.5%, 65.3%, 65.3%, and 65.3%, respectively (**Figure 2B**).

### 3.6 Subgroup analysis based on different combinations of camrelizumab-based therapies and treatment lines

PFS and OS were not different between patients who received camrelizumab combined with chemotherapy and those who received camrelizumab without chemotherapy. PFS and OS were also not different between patients who received camrelizumab combined with targeted therapy and those who received camrelizumab without targeted therapy (all *P*>0.05) (**Supplementary Figures 1A–D**). Additionally, it was also discovered that PFS (*P*=0.435) (**Supplementary Figure 2A**) and OS (*P*=0.833) (**Supplementary Figure 2B**) were not different in patients receiving different camrelizumab-based therapies, including camrelizumab monotherapy, camrelizumab plus chemotherapy, camrelizumab plus targeted therapy, camrelizumab plus chemotherapy and targeted therapy, camrelizumab plus chemotherapy and radiotherapy, and camrelizumab plus radiotherapy.

ORR was higher in patients receiving camrelizumab-based therapies as the first-line treatment than those receiving these therapies as the second or above-line treatment (*P*=0.014). Additionally, OS was prolonged in patients receiving camrelizumab-based therapies as the first-line treatment compared with those receiving these therapies as the second or above-line treatment (*P*=0.028) (**Supplementary Table 2**).

### 3.7 Cox regression model for PFS and OS

Regarding PFS, the univariable Cox regression analysis suggested that brain metastasis [hazard ratio (HR)=1.577, *P*=0.029] and liver metastasis (HR=1.775, *P*=0.027) were related to shorter PFS. According to the multivariable Cox regression analysis, brain metastasis (HR=1.548, *P*=0.036) and liver metastasis (HR=1.733, *P*=0.035) were independently associated with shorter PFS. The -2 Log Likelihood of the final Cox regression model of PFS was 1626.841, and the Chi-square value was 5.011 with a *P* value of 0.009, indicating a good fit to the data (**Table 4**).

**Table 4:** Univariable and multivariable analyses through Cox regression model for PFS and OS.

Characteristics	PFS				OS			
	*P* value	HR	Lower limit of 95%CI	Upper limit of 95%CI	*P* value	HR	Lower limit of 95%CI	Upper limit of 95%CI
Univariable analysis								
Age (≥65 years vs. <65 years)	0.097	0.773	0.571	1.048	0.362	0.759	0.419	1.374
Gender (male vs. female)	0.050	0.708	0.501	1.000	0.142	0.615	0.321	1.176
Smoking history (yes vs. no)	0.450	0.868	0.600	1.255	0.616	0.829	0.398	1.726
Drinking history (yes vs. no)	0.983	0.995	0.617	1.604	0.666	0.797	0.285	2.230
Previous lung diseases (yes vs. no)	0.780	0.898	0.421	1.913	0.959	1.038	0.251	4.290
Previous pulmonary surgery (yes vs. no)	0.366	1.177	0.827	1.675	0.787	0.904	0.434	1.881
Previous chemotherapy (yes vs. no)	0.253	1.210	0.873	1.677	0.034	2.215	1.062	4.617
Previous radiotherapy (yes vs. no)	0.470	0.879	0.620	1.247	0.282	1.409	0.755	2.631
Previous ICI therapy (yes vs. no)	0.384	1.329	0.701	2.521	0.481	1.525	0.472	4.928
ECOG PS score (>1 vs. 0~1)	0.051	1.474	0.998	2.179	0.070	1.920	0.948	3.890
Histological subtype								
Adenocarcinoma (reference)	Reference	(-)	(-)	(-)	Reference	(-)	(-)	(-)
Squamous cell carcinoma vs. reference	0.132	0.766	0.541	1.084	0.009	0.344	0.154	0.770
Small-cell carcinoma vs. reference	0.708	1.092	0.688	1.734	0.704	1.169	0.522	2.619
Others or UK vs. reference	0.566	0.846	0.477	1.499	0.921	0.952	0.362	2.506
Lymph node metastasis (yes vs. no)	0.249	0.836	0.616	1.134	0.495	0.812	0.447	1.476
Bone metastasis (yes vs. no)	0.164	1.307	0.897	1.907	0.210	1.572	0.775	3.188
Brain metastasis (yes vs. no)	0.029	1.577	1.049	2.370	0.005	2.675	1.346	5.319
Liver metastasis (yes vs. no)	0.027	1.775	1.067	2.954	0.033	2.604	1.079	6.284
Pleura metastasis (yes vs. no)	0.619	1.168	0.633	2.153	0.741	1.219	0.377	3.940
TNM stage								
IIIB (reference)	Reference	(-)	(-)	(-)	Reference	(-)	(-)	(-)
IIIC vs. reference	0.400	0.424	0.057	3.133	0.980	<0.001	<0.001	NA
IV vs. reference	0.216	1.308	0.854	2.004	0.366	1.489	0.629	3.526
*EGFR* mutation-positive (yes vs. no)	0.976	1.007	0.624	1.625	0.548	1.303	0.550	3.087
*ALK-*positive (yes vs. no)	0.871	1.177	0.164	8.452	0.763	0.049	<0.001	15733835.578
*ROS-1-*positive (yes vs. no)	0.871	1.177	0.164	8.452	0.763	0.049	<0.001	15733835.578
Camrelizumab combined with chemotherapy (yes vs. no)	0.199	0.818	0.602	1.112	0.563	0.837	0.459	1.528
Camrelizumab combined with targeted therapy (yes vs. no)	0.165	1.282	0.903	1.820	0.960	0.981	0.471	2.045
Multivariable analysis								
Previous chemotherapy (yes vs. no)	(-)	(-)	(-)	(-)	0.022	2.376	1.135	4.975
Brain metastasis (yes vs. no)	0.036	1.548	1.029	2.330	0.006	2.688	1.337	5.403
Liver metastasis (yes vs. no)	0.035	1.733	1.040	2.887	0.039	2.583	1.051	6.348

PFS, progression-free survival; OS, overall survival; HR, hazard ratio; CI, confidence interval; ICI, immune checkpoint inhibitors; ECOG PS, Eastern Cooperative Oncology Group Performance Status; UK, unknown; TNM, tumor-node-metastasis; NA, not available; EGFR, epidermal growth factor receptor; ALK, anaplastic lymphoma kinase; ROS-1, Ros proto-oncogene 1.

‘Sites of metastases’ and ‘Treatment lines’ were not included in the model: ‘Sites of metastases’ had a very strong association with ‘Lymph node metastasis,’ ‘Bone metastasis,’ ‘Brain metastasis,’’ Liver metastasis,’ ‘Pleura metastasis’; and ‘Treatment lines’ had a very strong association with ‘Previous chemotherapy’.

Regarding OS, the univariable Cox regression analysis showed that previous chemotherapy (HR=2.215, *P*=0.034), brain metastasis (HR=2.675, *P*=0.005), and liver metastasis (HR=2.604, *P*=0.033) were associated with shorter OS. Squamous cell carcinoma (vs. adenocarcinoma) was associated with prolonged OS (HR=0.344, *P*=0.009). Multivariable Cox regression analysis disclosed that previous chemotherapy (HR=2.376, *P*=0.022), brain metastasis (HR=2.688, *P*=0.006), and liver metastasis (HR=2.583, *P*=0.039) were independently associated with shorter OS. The -2 Log Likelihood of the final Cox regression model of OS was 415.235, and the Chi-square value was 18.630 with a *P* value of <0.001, indicating a good fit to the data (**Table 4**).

### 3.8 Adverse events

The median (range) onset time of adverse events was 1.2 (0.3-16.0) months. Common adverse events of any grade were reactive cutaneous capillary endothelial proliferation (RCCEP) (14.8%), pneumonia (6.0%), fatigue (5.4%), nausea and vomiting (4.4%), and gastrointestinal reaction (4.0%). Most adverse events were of grade I or II. Grade III and IV adverse events rarely occurred. Grade III adverse events included RCCEP (1.7%), myelosuppression (0.7%), gastrointestinal reaction (0.3%), and leukopenia (0.3%). Grade IV adverse events included myelosuppression (0.7%) and hypoalbuminemia (0.3%) (**Table 5**). Common camrelizumab-related adverse events included RCCEP (14.8%), fatigue (4.0%), pneumonia (2.3%), nausea and vomiting (2.3%), and fever (2.3%). Grade III camrelizumab-related adverse events included RCCEP (1.7%), myelosuppression (0.7%), gastrointestinal reaction (0.3%), and leukopenia (0.3%). No grade IV camrelizumab-related adverse events occurred (**Supplementary Table 3**). The management of grade I/II adverse events was guided by the Common Terminology Criteria for Adverse Events (CTCAE) and the Chinese Society of Clinical Oncology (CSCO) toxicity management guidelines. The management of grade III/IV adverse events involved suspending the treatment and addressing the situation in accordance with the toxicity management guidelines provided by the CSCO.

**Table 5:** Adverse events.

Events, n (%)	Any grade	Grade I	Grade II	Grade III	Grade IV
RCCEP	44 (14.8)	36 (12.1)	3 (1.0)	5 (1.7)	0 (0.0)
Pneumonia	18 (6.0)	11 (3.7)	7 (2.3)	0 (0.0)	0 (0.0)
Fatigue	16 (5.4)	16 (5.4)	0 (0.0)	0 (0.0)	0 (0.0)
Nausea and vomiting	13 (4.4)	13 (4.4)	0 (0.0)	0 (0.0)	0 (0.0)
Gastrointestinal reaction	12 (4.0)	11 (3.7)	0 (0.0)	1 (0.3)	0 (0.0)
Leukopenia	10 (3.4)	9 (3.0)	0 (0.0)	1 (0.3)	0 (0.0)
Myelosuppression	9 (3.0)	2 (0.7)	3 (1.0)	2 (0.7)	2 (0.7)
Fever	8 (2.7)	7 (2.3)	1 (0.3)	0 (0.0)	0 (0.0)
Anorexia	6 (2.0)	6 (2.0)	0 (0.0)	0 (0.0)	0 (0.0)
Cough	6 (2.0)	6 (2.0)	0 (0.0)	0 (0.0)	0 (0.0)
Thrombocytopenia	5 (1.7)	4 (1.3)	1 (0.3)	0 (0.0)	0 (0.0)
Hypoalbuminemia	3 (1.0)	1 (0.3)	1 (0.3)	0 (0.0)	1 (0.3)

RCCEP, reactive cutaneous capillary endothelial proliferation.

### 3.9 Comparison of clinical response and survival between patients with and without adverse events

DCR was different between patients with and without adverse events (*P*=0.003). In detail, 76.5% of patients without adverse events achieved DCR, and 89.8% of patients with adverse events achieved DCR. ORR, PFS, and OS were not different between patients with and without adverse events (all *P*>0.05) (**Supplementary Table 4**).

### 3.10 Subgroup analysis based on gender

PFS was longer in male patients than female patients (*P*=0.041). The median PFS (95% CI) was 11.0 (8.5-13.5) months in male patients; it was 7.0 (3.9-10.1) months in female patients. However, ORR (*P*=0.216), DCR (*P*=0.768), and OS (*P*=0.133) were not different between male and female patients (**Supplementary Table 5**).

### 3.11 Subgroup analysis based on different locations of Anhui province in China

All enrolled patients came from Anhui province. Therefore, the efficacy of camrelizumab-based therapies was compared among patients from Northern, Central, and Southern Anhui. ORR was different among patients from different locations in Anhui Province (*P*=0.020). ORR was 33.7% in patients from Northern Anhui, 29.5% in patients from Central Anhui, and 15.9% in patients from Southern Anhui. However, DCR (*P*=0.842), PFS (*P*=0.420), and OS (*P*=0.136) were not different among patients from Northern, Central, and Southern Anhui (**Supplementary Table 6**).

## 4 Discussion

Camrelizumab, alternatively named SHR-1210, effectively inhibits the interaction between PD-1 and PD-L1 and consequently suppresses the immune evasion of tumor cells (25). Several studies have reported that camrelizumab-based therapies achieve favorable treatment responses in patients with advanced lung cancer (21, 26–28). Of note, a real-world study observed that the ORR and DCR of camrelizumab-based therapies were 28.8% and 79.9% in patients with advanced NSCLC (27). Similarly to the previous study (27), we found that camrelizumab-based therapies achieved an ORR and DCR of 27.2% and 82.2%. However, by comparison with some phase 3 trials, although the DCR was similar, the ORR (64.8% (21) and 60.5% (23)) was higher in previous trials than that in our study. Potential reasons would be that (1): camrelizumab-based therapies were in the first-line setting in previous phase 3 trials (21, 23); however, our study did not restrict the treatment lines (2). The combined treatment strategy was camrelizumab plus chemotherapy in the previous phase trials (21, 23), but the combinations were diverse in our study (3). The histological subtype was squamous and non-squamous NSCLC in the previous phase 3 trials (21, 23), while our study included patients with various histological subtypes of lung cancer. These differences between our study and the previous phase 3 trials might contribute to the inconsistent results of ORR. Furthermore, we found that first-line camrelizumab-based therapies yielded a numerically higher ORR (36.6% vs. 22.9%) compared to second or subsequent-line camrelizumab-based therapies. Our findings supported that camrelizumab-based therapies possessed the potential to serve as the first-line treatment for advanced lung cancer.

Driver gene alterations could contribute to the resistance to ICIs, but patients with some specific driver gene alterations could benefit from ICIs (29). As reported by a previous study, patients with advanced NSCLC harboring *B-raf proto-oncogene (BRAF)* V600E mutation showed a superior benefit from ICIs (30). In our study, we observed that ORR and DCR were not affected by *EGFR* mutation-positive*, ALK*-positive*, and ROS-1*-positive in patients with advanced lung cancer who received camrelizumab-based therapies. However, only 10.4%, 1.3%, and 1.3% of patients carried *EGFR* mutation-positive*, ALK*-positive*, and ROS-1*-positive in this study, which might affect the statistical power. Therefore, our findings should be validated by more studies.

Considering that advanced lung cancer predominantly affects the older, and aging is related to a decline in immune function, it is meaningful to investigate whether the older patients could benefit from camrelizumab-based therapies (31–33). A previous study found that the ORR of camrelizumab-based therapies was not affected by age ≥70 years or <70 years in patients with advanced NSCLC (28). In this research, we applied 65 as the cutoff value and found that age ≥65 years was positively associated with ORR. Our findings provided a reference that camrelizumab-based therapies could be recommended for patients with advanced lung cancer aged more than 65 years.

A previous study found that treatment line, liver metastasis, and treatment duration were strong factors associated with ORR in patients with advanced NSCLC who received camrelizumab-based therapies (28). Our study observed that previous pulmonary surgery, previous radiotherapy, and ECOG PS score >1, were independently and negatively associated with ORR. The potential reasons would be that: (1) previous pulmonary surgery or radiotherapy might affect the tumor microenvironment, which facilitated the immune escape of tumor cells and compromised the efficacy of camrelizumab-based therapies (34–36). (2) An ECOG PS score >1 signified a higher level of impaired physical performance, which could attenuate patients’ ability to tolerate camrelizumab-based therapies, ultimately limiting the therapeutic benefit (37, 38).

Camrelizumab-based therapies achieve satisfactory survival profiles in patients with advanced lung cancer according to previous studies (21, 23, 27, 28). The CameL study reported that camrelizumab plus chemotherapy resulted in the median PFS (95% CI) of 11.0 (8.5-12.5) months and the median OS (95% CI) of 27.1 (21.9-31.5) months in patients with advanced non-squamous NSCLC (26). Consistent with the previous study (26), we found that the median PFS (95% CI) was 10.0 (7.8-12.2) months. However, the median OS was not achieved in our study due to the short follow-up duration. In addition, a previous study reported that the 12-month PFS and OS rates were 50.1% and 73.4% in patients with advanced NSCLC (27). Similarly to the finding of this previous study (27), we found that the 12-month PFS and OS rates were 41.2% and 78.5%. Moreover, we discovered that the 24-, 36-, and 48-month OS rates were 65.3%. However, these data should be interpreted with caution since the median follow-up duration was only 5 months in this study. Further studies with extended follow-up duration were warranted to assess the survival outcomes in patients with advanced lung cancer who received camrelizumab-based therapies.

As reported by previous studies, histological subtype, treatment lines, camrelizumab treatment duration, liver metastasis, and brain metastasis could affect the survival profiles of patients with advanced lung cancer who received ICIs-based therapies (27, 28, 39, 40). Consistent with previous studies (28, 39, 40), we found that brain metastasis and liver metastasis were independently linked with both shorter PFS and OS in patients with advanced lung cancer who received camrelizumab-based therapies. A potential reason would be that brain metastasis and liver metastasis represented an aggressive manifestation of the disease, which might restrain the efficacy of camrelizumab-based therapies and ultimately lead to poor survival (39). In clinical practice, the combinations of camrelizumab-based therapies for the treatment of advanced lung cancer exhibit marked diversity (41, 42), and whether patients could benefit from specific combinations deserves to be explored. According to a previous study, survival profiles were not influenced by combinations in patients with advanced NSCLC who received camrelizumab-based therapies (27). Consistent with the previous study (27), we also found that PFS and OS were not affected by different combinations of camrelizumab-based therapies. Our findings suggested that physicians could consider different treatments in combination with camrelizumab according to patients’ actual conditions. It should be clarified that the classification of the treatment regimen was relatively rough in this study. Therefore, a controlled study with standardized treatment arms was required to reduce the variability and strengthen efficacy assessments for each regimen.

Currently, the findings concerning the impact of gender on the efficacy of immunotherapy are inconsistent (43, 44). In our study, we found that male patients with advanced lung cancer benefited more from camrelizumab-based therapies than female patients in terms of survival. We speculated that some differences between male and female patients, such as sex hormones, genetic factors, and immune response to lung cancer, may contribute to different survival after camrelizumab-based therapies (45, 46). However, the numbers of male and female patients are largely different in our study, which might affect the statistical power. Therefore, the impact of sex on camrelizumab-based therapies in patients with advanced lung cancer should be further validated. Moreover, we also discovered that ORR was different among patients from Northern, Central, and Southern Anhui. Varying levels of medical care in different locations of Anhui might lead to differences in the efficacy of camrelizumab-based therapies in patients with advanced lung cancer.

Safety is one of the major concerns regarding the application of camrelizumab-based therapies for the treatment of advanced lung cancer. Previous studies have reported that common adverse events include decreased white blood cell count, decreased neutrophil count, anemia, and RCCEP in patients with advanced lung cancer who received camrelizumab-based therapies (21, 23, 27). In the current study, we found that the common adverse events at any grade were RCCEP, pneumonia, fatigue, nausea and vomiting, and gastrointestinal reactions in patients with advanced lung cancer who received camrelizumab-based therapies. Overall, most adverse events were mild and manageable, suggesting that camrelizumab-based therapies were safe for advanced lung cancer. Notably, the incidence of RCCEP (14.8%) in our study was relatively lower than that of previous studies (ranging from 18.6% to 78.0%) (21, 23, 27). An explanation was that in this study, some patients received pre-treatment for RCCEP with thalidomide, leading to a relatively low incidence of RCCEP. However, the use of thalidomide to prevent the occurrence of RCCEP was based on the clinical experience in some study centers. Therefore, we did not record the specific number of patients who used thalidomide to prevent the occurrence of RCCEP. To support the clinical practice, future research might be needed to explore the role of preventative thalidomide use in reducing RCCEP. Of note, several previous studies suggested that adverse events were related to the good efficacy of ICIs in patients with advanced lung cancer (47–49). In line with these previous studies, we also observed that adverse events were positively associated with DCR. A potential explanation would be that patients who experienced adverse events might have a more competent immune system, contributing to a higher likelihood of responding to camrelizumab-based therapies (50).

Several limitations should be noted in the current study. (1) Our study shared real-world experience of applying camrelizumab-based therapies to treat patients with advanced lung cancer. Nevertheless, the research region was limited to Anhui province in China, and our findings should be further verified by studies involving diverse geographic and ethnic populations. (2) The single-arm, observational design of this study might induce selection bias and hinder us from drawing a solid conclusion about the superior efficacy of camrelizumab-based therapies versus other treatments in patients with advanced lung cancer. Therefore, further studies could consider designing randomized, controlled trials or comparative observational studies to validate the efficacy and safety of camrelizumab for the treatment of advanced lung cancer. (3) The median follow-up duration was only 5 months, which was relatively short for assessing OS and long-term safety. Considering that the effect of camrelizumab on improving survival required a long period due to delayed immune responses, further studies with long-term follow-up duration were warranted to validate the survival benefits and durability of camrelizumab-based therapies in patients with advanced lung cancer. (4) Although our subgroup analysis found that PFS and OS were not different in patients receiving different camrelizumab-based therapies, the sample size was small in some subgroups, which might affect the statistical power. Therefore, further studies with large sample sizes were required to validate our findings. (5) The number of patients who carried driver gene mutations was small. Therefore, the impact of driver gene mutations on the efficacy of camrelizumab-based therapies should be further verified. (6) Several subgroup analyses based on gender, treatment lines, and geographic locations were conducted. Nevertheless, they might be underpowered due to the small sample size in some subgroups. Therefore, the findings of our subgroup analyses should be further validated by studies with large sample sizes or pooled analyses.

In summary, camrelizumab-based therapies achieve good efficacy with tolerable safety profiles in patients with advanced lung cancer. The efficacy of first-line camrelizumab-based therapies is profound compared with the second or above-line setting in patients with advanced lung cancer. Our findings provide real-world evidence that camrelizumab-based therapies may be a potential first-line treatment for patients with advanced lung cancer.

## Data Availability

The original contributions presented in the study are included in the article/**Supplementary Material**. Further inquiries can be directed to the corresponding author.
