# Supplementary material for: Camrelizumab-based therapies for the treatment of advanced lung cancer: a prospective, open-label, multicenter, observational, real-world study
Source: Front Immunol. 2025 Mar 7;16:1494708. doi: 10.3389/fimmu.2025.1494708 (PMC11925761; doi:10.3389/fimmu.2025.1494708)
Supplement: Supplementary file 1 [file DataSheet1.docx]

***Supplementary Materials***

**
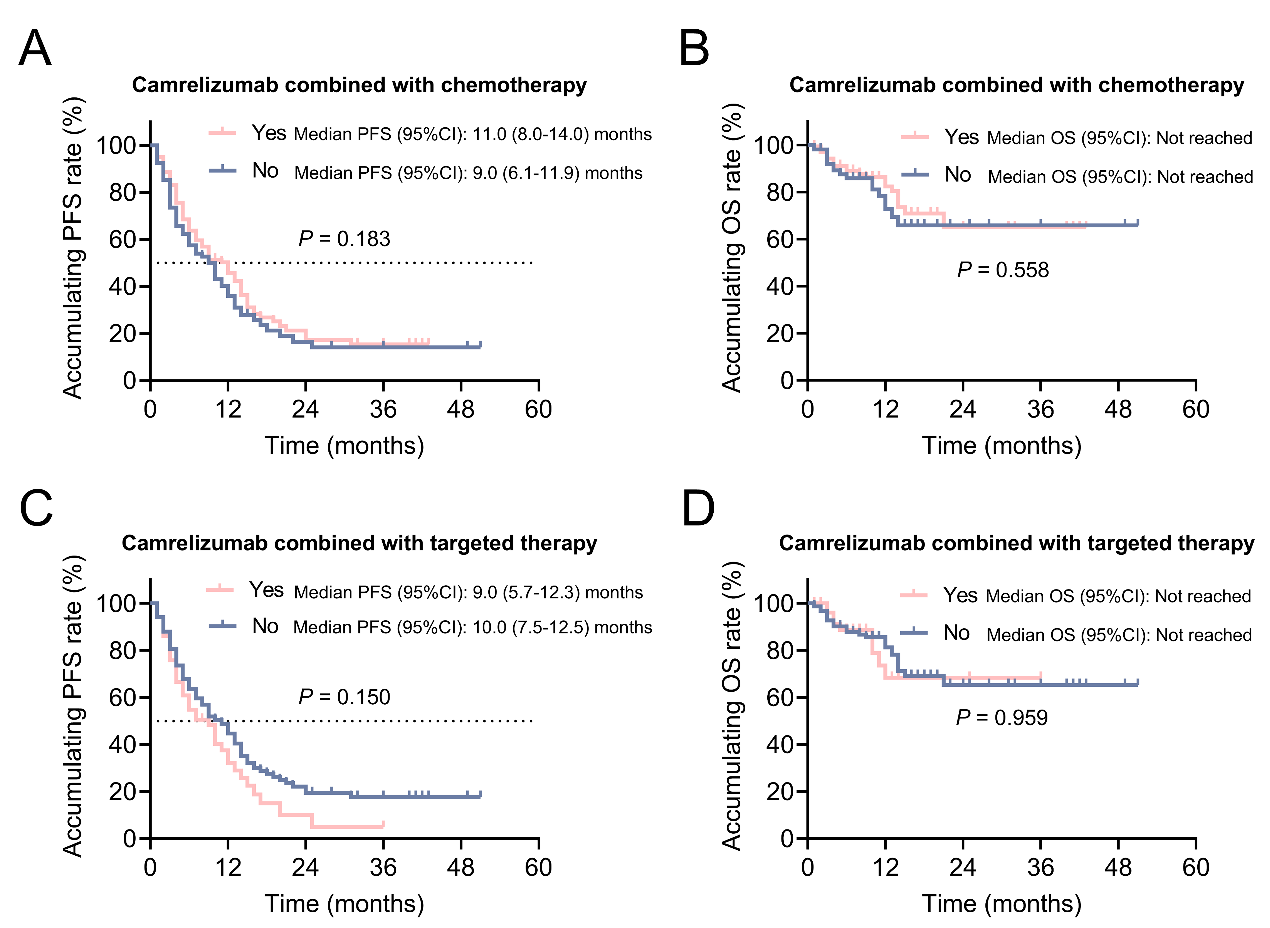
**

**Supplementary Figure 1.** Subgroup analysis for PFS and OS based on different camrelizumab-based therapies in patients with advanced lung cancer. Comparison of PFS (**A**) and OS (**B**) between patients with advanced lung cancer who received camrelizumab combined with chemotherapy and those who received camrelizumab without chemotherapy. Comparison of PFS (**C**) and OS (**D**) between patients with advanced lung cancer who received camrelizumab combined with targeted therapy and those who received camrelizumab without targeted therapy.

**
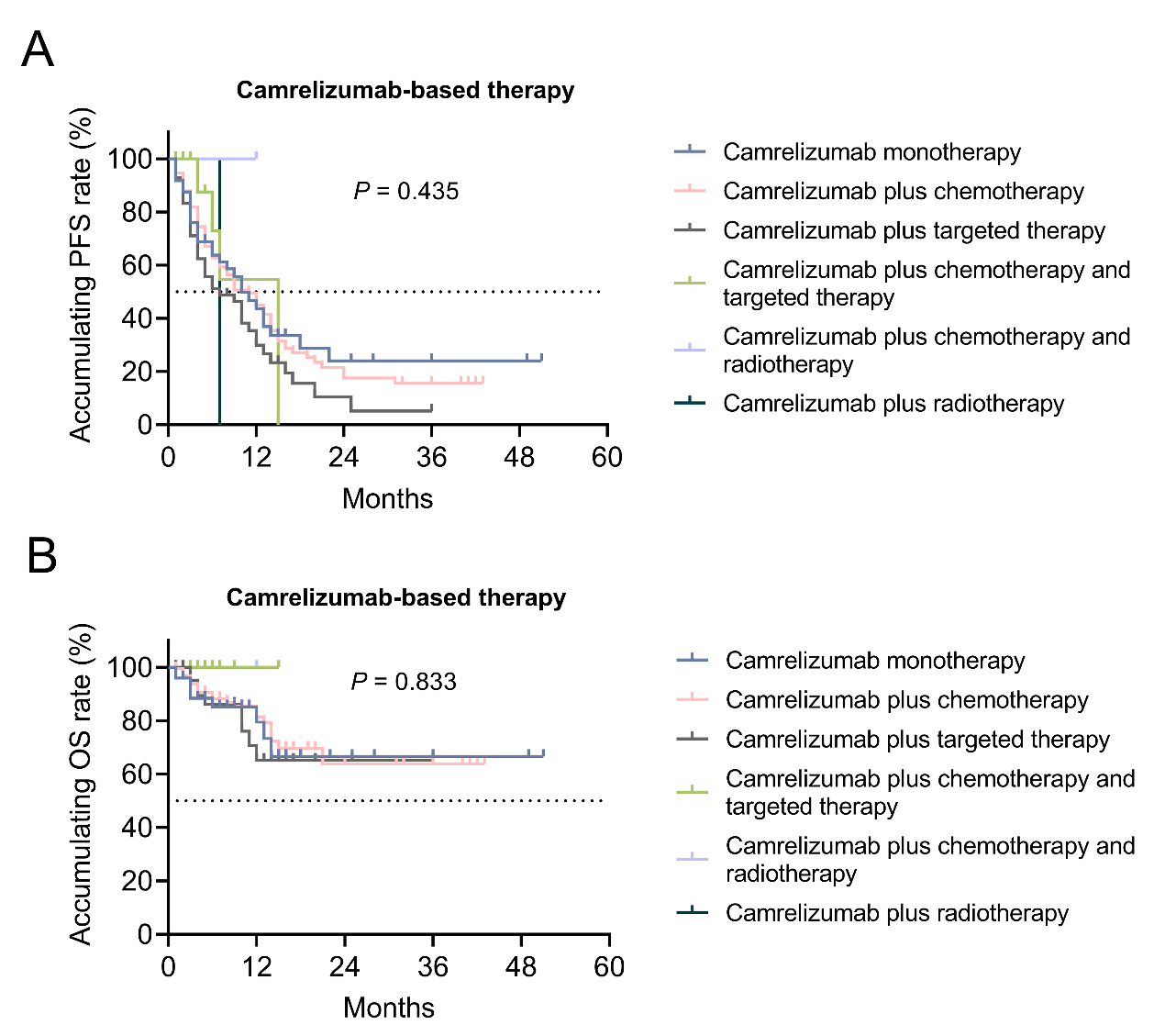
**

**Supplementary Figure 2.** Subgroup analysis for PFS and OS among patients with advanced lung cancer receiving 6 different camrelizumab-based therapies. Comparison of PFS (**A**) and OS (**B**) among patients with advanced lung cancer receiving 6 different camrelizumab-based therapies.

**Supplementary Table 1.** Association between *EGFR* mutation-, *ALK-*, *ROS-1-*positive and clinical response.

| Items | ORR | *P* value | DCR | *P* value |
| --- | --- | --- | --- | --- |
| *EGFR* mutation-positive, n (%) |  | 0.502 |  | 0.461 |
| No (n = 267) | 71 (26.6) |  | 221 (82.8) |  |
| Yes (n = 31) | 10 (32.3) |  | 24 (77.4) |  |
| *ALK-*positive, n (%) |  | 0.063 |  | 1.000 |
| No (n = 294) | 78 (26.5) |  | 241 (82.0) |  |
| Yes (n = 4) | 3 (75.0) |  | 4 (100.0) |  |
| *ROS-1-*positive, n (%) |  | 0.063 |  | 1.000 |
| No (n = 294) | 78 (26.5) |  | 241 (82.0) |  |
| Yes (n = 4) | 3 (75.0) |  | 4 (100.0) |  |

EGFR, epidermal growth factor receptor; ALK, anaplastic lymphoma kinase; ROS-1, Ros proto-oncogene 1; ORR, objective response rate; DCR, disease control rate.

**Supplementary Table 2. Comparison of ORR, DCR, PFS, and OS between patients with different treatment lines.**

| Items | First-line  (n = 93) | Second or above-line  (n = 205) | *P* value |
| --- | --- | --- | --- |
| ORR, n (%) |  |  | 0.014 |
| No | 59 (63.4) | 158 (77.1) |  |
| Yes | 34 (36.6) | 47 (22.9) |  |
| DCR, n (%) |  |  | 0.247 |
| No | 13 (14.0) | 40 (19.5) |  |
| Yes | 80 (86.0) | 165 (80.5) |  |
| PFS (months) |  |  | 0.236 |
| Median PFS | 12.0 | 8.0 |  |
| 95% CI | 8.4-15.6 | 5.3-10.7 |  |
| 12-month PFS rate | 49.4% | 37.3% | (-) |
| 24-month PFS rate | 13.9% | 18.1% | (-) |
| 36-month PFS rate | 10.4% | 16.6% | (-) |
| OS (months) |  |  | 0.028 |
| Median OS | Not reached | Not reached |  |
| 95% CI | (-) | (-) |  |
| 12-month OS rate | 88.6% | 73.7% | (-) |
| 24-month OS rate | 74.9% | 61.3% | (-) |
| 36-month OS rate | 74.9% | 61.3% | (-) |

ORR, objective response rate; DCR, disease control rate; PFS, progression-free survival; OS, overall survival; CI, confidence interval.

**Supplementary Table 3. Camrelizumab-related adverse events.**

| Events, n (%) | Any grade | Grade I | Grade II | Grade III | Grade IV | NA |
| --- | --- | --- | --- | --- | --- | --- |
| RCCEP | 44 (14.8) | 16 (5.4) | 2 (0.7) | 5 (1.7) | 0 (0.0) | 21 (7.0) |
| Pneumonia | 7 (2.3) | 0 (0.0) | 1 (0.3) | 0 (0.0) | 0 (0.0) | 6 (2.0) |
| Fatigue | 12 (4.0) | 7 (2.3) | 0 (0.0) | 0 (0.0) | 0 (0.0) | 5 (1.7) |
| Nausea and vomiting | 7 (2.3) | 4 (1.3) | 0 (0.0) | 0 (0.0) | 0 (0.0) | 3 (1.0) |
| Gastrointestinal reaction | 3 (1.0) | 2 (0.7) | 0 (0.0) | 1 (0.3) | 0 (0.0) | 0 (0.0) |
| Leukopenia | 6 (2.0) | 2 (0.7) | 0 (0.0) | 1 (0.3) | 0 (0.0) | 3 (1.0) |
| Myelosuppression | 6 (2.0) | 0 (0.0) | 3 (1.0) | 2 (0.7) | 0 (0.0) | 1 (0.3) |
| Fever | 7 (2.3) | 3 (1.0) | 1 (0.3) | 0 (0.0) | 0 (0.0) | 3 (1.0) |
| Anorexia | 4 (1.3) | 2 (0.7) | 0 (0.0) | 0 (0.0) | 0 (0.0) | 2 (0.7) |
| Cough | 3 (1.0) | 1 (0.3) | 0 (0.0) | 0 (0.0) | 0 (0.0) | 2 (0.7) |
| Thrombocytopenia | 5 (1.7) | 0 (0.0) | 1 (0.3) | 0 (0.0) | 0 (0.0) | 4 (1.3) |
| Hypoalbuminemia | 2 (0.7) | 0 (0.0) | 1 (0.3) | 0 (0.0) | 0 (0.0) | 1 (0.3) |

RCCEP, reactive cutaneous capillary endothelial proliferation; NA, not available.

**Supplementary Table 4.** Comparison of ORR, DCR, PFS, and OS between patients with and without adverse events.

| Items | Without adverse events  (n = 170) | With adverse events  (n = 128) | *P* value |
| --- | --- | --- | --- |
| ORR, n (%) |  |  | 0.751 |
| No | 125 (73.5) | 92 (71.9) |  |
| Yes | 45 (26.5) | 36 (28.1) |  |
| DCR, n (%) |  |  | 0.003 |
| No | 40 (23.5) | 13 (10.2) |  |
| Yes | 130 (76.5) | 115 (89.8) |  |
| PFS (months) |  |  | 0.209 |
| Median PFS | 8.0 | 12.0 |  |
| 95% CI | 5.2-10.8 | 8.6-15.4 |  |
| OS (months) |  |  | 0.331 |
| Median OS | Not reached | Not reached |  |
| 95% CI | (-) | (-) |  |

ORR, objective response rate; DCR, disease control rate; PFS, progression-free survival; OS, overall survival; CI, confidence interval.

**Supplementary Table 5.** Comparison of ORR, DCR, PFS, and OS between male and female patients.

| Items | Male  (n = 235) | Female  (n = 63) | *P* value |
| --- | --- | --- | --- |
| ORR, n (%) |  |  | 0.216 |
| No | 175 (74.5) | 42 (66.7) |  |
| Yes | 60 (25.5) | 21 (33.3) |  |
| DCR, n (%) |  |  | 0.768 |
| No | 41 (17.4) | 12 (19.0) |  |
| Yes | 194 (82.6) | 51 (81.0) |  |
| PFS (months) |  |  | 0.041 |
| Median PFS | 11.0 | 7.0 |  |
| 95% CI | 8.5-13.5 | 3.9-10.1 |  |
| 12-month PFS rate | 44.4% | 31.1% | (-) |
| 24-month PFS rate | 18.8% | 12.6% | (-) |
| 36-month PFS rate | 17.1% | 6.3% | (-) |
| OS (months) |  |  | 0.133 |
| Median OS | Not reached | Not reached |  |
| 95% CI | (-) | (-) |  |
| 12-month OS rate | 81.0% | 69.4% | (-) |
| 24-month OS rate | 69.7% | 51.2% | (-) |
| 36-month OS rate | 69.7% | 51.2% | (-) |

ORR, objective response rate; DCR, disease control rate; PFS, progression-free survival; OS, overall survival; CI, confidence interval.

**Supplementary Table 6.** Comparison of ORR, DCR, PFS, and OS among patients from different locations of Anhui province in China.

| Items | Southern Anhui  (n = 82) | Central Anhui  (n = 112) | Northern Anhui  (n = 104) | *P* value |
| --- | --- | --- | --- | --- |
| ORR, n (%) |  |  |  | 0.020 |
| No | 69 (84.1) | 79 (70.5) | 69 (66.3) |  |
| Yes | 13 (15.9) | 33 (29.5) | 35 (33.7) |  |
| DCR, n (%) |  |  |  | 0.824 |
| No | 15 (18.3) | 18 (16.1) | 20 (19.2) |  |
| Yes | 67 (81.7) | 94 (83.9) | 84 (80.8) |  |
| PFS (months) |  |  |  | 0.420 |
| Median PFS | 8.0 | 11.0 | 9.0 |  |
| 95% CI | 4.8-11.2 | 7.6-14.4 | 5.0-13.0 |  |
| 12-month PFS rate | 34.4% | 44.6% | 41.9% | (-) |
| 24-month PFS rate | 141% | 21.8% | 11.5% | (-) |
| 36-month PFS rate | 14.1% | 19.4% | 7.7% | (-) |
| OS (months) |  |  |  | 0.136 |
| Median OS | 21.0 | Not reached | Not reached |  |
| 95% CI | 6.8-35.2 | (-) | (-) |  |
| 12-month OS rate | 70.8% | 84.3% | 77.2% | (-) |
| 24-month OS rate | 43.4% | 74.9% | 69.2% | (-) |
| 36-month OS rate | 43.4% | 74.9% | 69.2% | (-) |

ORR, objective response rate; DCR, disease control rate; PFS, progression-free survival; OS, overall survival; CI, confidence interval.
